# Proteomics Reveals Octyl Gallate as an Environmentally Friendly Wood Preservative Leading to Reactive Oxygen Species-Driven Metabolic Inflexibility and Growth Inhibition in White-Rot Fungi (*Lenzites betulina* and *Trametes versicolor*)

**DOI:** 10.3390/jof7020145

**Published:** 2021-02-17

**Authors:** Jin-Wei Xu, Chen-Chung Liao, Ke-Chang Hung, Zhong-Yao Wang, Yu-Tang Tung, Jyh-Horng Wu

**Affiliations:** 1 Department of Forestry, National Chung Hsing University, Taichung 40227, Taiwan; ecsgunro@gmail.com (J.-W.X.); d9833004@mail.nchu.edu.tw (K.-C.H.); hi4u2ca707@gmail.com (Z.-Y.W.); 2 Proteomics Research Center, National Yang-Ming University, Taipei 11221, Taiwan; ccliao@ym.edu.tw; 3 Graduate Institute of Biotechnology, National Chung Hsing University, Taichung 40227, Taiwan

**Keywords:** antifungal activity, white-rot fungus, octyl gallate, proteomics, mitochondrial dysfunction, wood preservative

## Abstract

The most commonly applied wood preservatives are based on creosote, pentachlorophenol, and waterborne chromate copper arsenate, which negatively affect the environment. Thus, environmentally friendly wood preservatives are required. This study investigated the antifungal activity and mechanism of several long-chain alkyl gallates (3,4,5-trihydroxybenzoates) against white-rot fungi, *Lenzites betulina* and *Trametes versicolor*. The results revealed that octyl gallate (OG) had the best antifungal activity. Additionally, OG may have a mechanism of action similar to surfactants and inhibit ATPase activity, causing mitochondrial dysfunction and endogenous reactive oxygen species (ROS) production. Upon exposure to endogenous ROS, cells rapidly inhibit the synthesis of 60S ribosomal subunits, thus reducing the mycelial growth rate. *L. betulina* and *T. versicolor* also remodeled their energy metabolism in response to low ATP levels and endogenous ROS. After OG treatment, ATP citrate synthase activity was downregulated and glycolytic activity was upregulated in *L. betulina*. However, the activity of aerobic pathways was decreased and the oxidative branch of the pentose phosphate pathway was redirected form nicotinamide adenine dinucleotide phosphate (NADPH) to minimize endogenous ROS-mediated damage in *T. versicolor*. Taken together, these observations reveal that OG is a potent inhibitor of white-rot fungus. Further structural optimization research and pharmacological investigations are warranted.

## 1. Introduction

Wood is widely used for architecture, decoration, and furniture because it is inexpensive and renewable and has low density, high impact strength, and low thermal conduction. However, wood is easily damaged by various biodeteriogens, such as fungi, bacteria, termites, insects, and marine microorganisms [1]. In general, wood degradation causes a loss of billions of dollars (USD) every year to repair and replace the damaged wood structures [2]. To prolong its lifespan, wood is commonly treated with preservatives to achieve protection against a broad range of biodeteriogens. The most commonly applied wood preservatives are creosote, pentachlorophenol, and waterborne chromate copper arsenate (CCA), because of their high degree of toxicity to fungi and insects [3,4]. However, they have negative effects on the environment. The chromium and arsenic leaching from CCA are known to be potent carcinogens. Therefore, CCA has been withdrawn from residential applications. Research has focused on environmentally friendly preservatives, and many extractives and agricultural waste products have been identified as potential inhibitors of wood degradation, including linseed and tung oil, tannins, stilbenes, methanolic extractives of beech wound-wood, pyrolysis distillates, and spent coffee grounds [5]. Among them, extractives and tannins with antifungal and insecticidal properties have attracted the most attention [6].

In many tannins, octyl gallate (OG) not only exerts diverse biological effects besides its antioxidant activity such as antibacterial, antifungal, antiviral, antidiabetic, anti-amyloidogenic, and anticancer effects [7], but also exhibits activity against wood-decay fungi such as *Lenzites betulina*, *Trametes versicolor*, and *Gloeophyllum trabeum* [8]. Adding gallate derivatives to biocide reduces its inhibitory concentration values 2–256 times [9]. Gallate derivatives are also found in many phytomedicines. They have diverse biological and pharmacological activities, including free radical scavenging, maintenance of endogenous defense systems, metal ion chelation, inhibition of cell signaling pathways, and cancer cell apoptosis [10]. They may exert antifungal activity by disrupting the native membrane-associated function of the integral proteins as nonionic surfactants rather than common antioxidants [11,12]. However, specific studies on their antifungal activity against basidiomycete white-rot fungi are limited. Moreover, the role of hydrophobic alkyl chain length on gallate derivatives remains poorly understood. Therefore, this study investigated the antifungal activity of a series of long-chain alkyl gallates (3,4,5-trihydroxybenzoates) against the most common and worldwide distributed white-rot fungi (*L. betulina* and *T. versicolor*) using the agar dilution method. Additionally, laccase and manganese peroxidase (MnP) assays and proteomics analysis were conducted to identify changes in *L. betulina* and *T. versicolor* in response to the addition of OG.

## 2. Materials and Methods

### 2.1. Chemicals and Fungal Strain

Gallic acid, methyl gallate (MG), ethyl gallate (EG), butyl gallate (BG), OG, decyl gallate (DG), and hexadecyl gallate (HG) were purchased from Tokyo Kasei Kogyo (Tokyo, Japan). The other chemicals and solvents used in this experiment were purchased from Merck (Darmstadt, Germany). The white-rot fungi *L. betulina* (Fries) (BCRC35296) and *T. versicolor* (Linnaeus: Fries) (BCRC35253) were obtained from the Bioresource Collection and Research Center of Food Industry Research and Development Institute in Taiwan.

### 2.2. Antifungal Assays

The agar dilution method was used to evaluate the antifungal activity of long-chain alkyl gallates following the method reported by Hsu et al. [7]. The long-chain alkyl gallates were dissolved in 15 μL of methanol, which was added to 5 mL of sterilized potato dextrose agar solution (PDA, 39 g/L) in a 6-cm petri dish. After the solidification of the media, *L. betulina* and *T. versicolor* plugs (3 mm in diameter) were placed on PDA media, and the petri dish was sealed with parafilm and incubated at 27 ± 2 °C and 70% relative humidity (RH). When the mycelia reached the edges of control plates (methanol was used as the control), antifungal assays were performed. The antifungal index (%) was calculated as follows: (1 − D*t*/D*c*) × 100, where D*t* and D*c* are growth zone diameters (cm) in the experimental and control plates, respectively.

Submerged cultivation was used to evaluate antifungal and enzyme activities following the method reported by Kubo et al. [11] with slight modifications. The OG was dissolved in 1 mL of methanol, which was added to 150 mL of sterilized malt extract solution (17 g/L) in a 250-mL flask. When the media returned to 27 °C, a mycelial plug (3 mm in diameter) was placed on the media. The flask was sealed with parafilm and incubated at 150 rpm, 27 ± 2 °C, and 70% RH. In brief, incubation times of 9 and 7 days for *L. betulina* and *T. versicolor*, respectively, were selected when the weight of growing mycelia reached 50 mg. When mycelia reached 50 mg in weight in the control flask (methanol was used as the control), the weight of mycelium and the pH of the filtrate were recorded. Antifungal assays were performed three times. The antifungal index (%) was calculated as (1 − W*t*/W*c*) × 100, where W*t* and W*c* represent the weight (mg) of mycelia in the experimental and control flasks, respectively.

### 2.3. 1,1-Diphenyl-2-Picrylhydrazyl Assay

The 1,1-diphenyl-2-picrylhydrazyl (DPPH) radical-scavenging activity of long-chain alkyl gallates was examined according to the method reported by Lin et al. [13]. In brief, 10 μL of compounds in DMSO (final concentrations of 1, 5, 10, 25, 50, and 100 μg/mL) were mixed with 90 μL of 50 mM Tris–HCl buffer (pH 7.4) and 200 μL of a 0.1 mM DPPH–ethanol solution. After 30 min of incubation at ambient temperature, the reduction in DPPH free radicals was determined according to the absorbance at 517 nm, which was measured using the ELISA reader. (+)-Catechin was used as a positive control. Three replicates were made for each test sample. The inhibition ratio (%) was calculated as (1 − A*t*/A*c*) × 100, where A*t* is the absorbance of the experimental reaction and A*c* is the absorbance of the control reaction.

### 2.4. Ferrous Ion-Chelating Ability Assay

The ferrous ion-chelating potential of long-chain alkyl gallates was evaluated following the method reported by Lin et al. [13]. In brief, 200 μL of a series of long-chain alkyl gallates in methanol (final concentrations of 0.1, 0.2, 0.5, 1, 2, 5, 10, 20, 50, 100, 200, 500, and 1000 μg/mL) and 740 μL of methanol were added to 20 μL of 2 mM FeCl_2_. The reaction was initiated through the addition of 40 μL of 5 mM ferrozine. The mixture was shaken vigorously and allowed to rest at ambient temperature for 10 min. Absorbance of the solution was measured at 562 nm. Ethylenediaminetetraacetic acid (EDTA) was used as a positive control. Three replicates were made for each test sample. The percentage of inhibition of the ferrozine–Fe^2+^ complex formation was calculated as (1 − A*t*/A*c*) × 100, where A*t* is the absorbance of the experimental reaction and A*c* is the absorbance of the control reaction.

### 2.5. Determination of Laccase and Manganese Peroxidase Activities

This assay was determined according to the method described by Tamagawa et al. [14]. The laccase activity was determined through monitoring of the oxidation of 2,6-dimethoxyphenol (DMP) at 470 nm and 30 °C. The solution (300 μL) was mixed with 1 mM DMP and 50 mM malonate buffer (pH 4.5). The manganese peroxidase (MnP) activity was determined in the same manner, except that the reaction mixture also contained 0.1 mM MnSO_4_ and 0.2 mM H_2_O_2_.

### 2.6. Protein Sample Preparation

Each mycelium sample from submerged cultivation was placed in a 1.5-mL sample tube that contained 0.2 g of ceramic beads (0.5 mm in diameter), 5 μL of phenylmethylsulfonyl fluoride, and 0.25 mL of breaking buffer (0.69 g of sodium phosphate, 0.037 g of EDTA, 5 mL of glycerol, and 90 mL of deuterium-depleted water). Tissue debris was removed through centrifugation at 12,000× *g* for 10 min at 4 °C, and the supernatant was transferred to a new Eppendorf tube. The protein concentration was measured using a bicinchoninic acid protein assay kit (Thermo, Rockford, IL, USA).

### 2.7. SDS-PAGE

Mycelium protein samples were fractionated by 12% SDS-PAGE (Hoefer^®^, Holliston, MA, USA) according to the method described by Liao et al. [15]. In brief, 50 μg of each protein sample was applied to the gel in triplicate, and the sizes of proteins were visualized through staining with Coomassie Brilliant Blue. The gel lanes were split into 10 equal fractions, and the slices were destained through repeat washing in a solution of 25 mM ammonium bicarbonate and 50% (*v*/*v*) acetonitrile (1:1) until the protein bands were invisible. The resulting gel was dried in a SpeedVac and stored at −20 °C.

### 2.8. Nanoflow Ultra-High-Performance Liquid Chromatography−Tandem Mass Spectrometry

Each cryostored tryptic digest was resuspended in 30 μL of 0.1% (*v*/*v*) formic acid and analyzed using an online nanoAcquity ultra-performance liquid chromatography (UPLC) system (Waters, Manchester, UK) coupled to a hybrid linear ion trap Orbitrap (LTQ-Orbitrap Discovery) mass spectrometer with a nanoelectrospray ionization source (Thermo Scientific, San Jose, CA, USA). After the sample was loaded with a single injection model into the UPLC, the peptides were captured and desalted on a C18 trap column (180 μm × 20 mm; Waters, MA, USA) and then further separated using a BEH C18 column (25 cm × 75 µm; Waters, MA, USA). Mobile phase solvents A and B were prepared as 0.1% formic acid in water and 0.1% formic acid in acetonitrile, respectively. The separation condition was achieved through elution of the peptides from the column with a linear gradient of 5–35% A/B for 90 min, 35–95% A/B for 2 min, and 95% A/B for 10 min at a flow rate of 0.5 μL/min. The eluted peptides were ionized with a spray voltage of 2.33 kV and introduced into the mass spectrometer. Mass spectrometric data were obtained using a data-dependent acquisition method (isolation width: 2 Da) in which one full MS survey scan (m/z: 200–1500) at a high resolution of 30,000 full at half maximum width was followed by tandem mass spectrometry (MS/MS) scanning (m/z: 200–1500) of the six most intense multiply charged ions (2^+^ and 3^+^). Fragment ions of each selected precursor were generated through collision-induced dissociation using helium gas with collision energy of 35% (or 3.5 eV). The dynamic exclusion duration of precursors was set to 120 s with an exclusion list size of 200.

### 2.9. Mass Spectrometric Data Analysis

Liquid chromatography–MS/MS raw data were analyzed with Peaks 7.5 Studio software for proteomics (Bioinformatics Solutions, Waterloo, Canada). The search was conducted against the UniProt database (containing 1,715,780 protein sequences; released on January 2020; http://www.uniprot.org/; accessed on 29 September 2020). The search parameters were as follows: parent mass error tolerance, 50 ppm; fragment mass error tolerance, 0.8 Da; enzyme set as trypsin; and two missed cleavages allowed, with oxidation on methionine (+15.99 Da) and carbamidomethylation on cysteine (+57.02 Da) used as variable modifications. The average local confidence was established at > 80%. A decoy database was used to calculate the false discovery rate, which was set to < 1%. A protein was identified when at least one unique peptide was matched. Protein quantitative analysis using MS spectra counting involved the use of in-house software [16,17].

### 2.10. Statistical Analysis

All results were expressed as the mean ± standard deviation. The significance of differences was calculated using Scheffe’s test, and values of < 0.05 were considered to be significant. This method made all pairwise comparisons between the means and was a very reliable procedure that violated the assumptions associated with ANOVA.

## 3. Results and Discussion

### 3.1. Antifungal Activity of Long-Chain Alkyl Gallates

**Figure 1:**
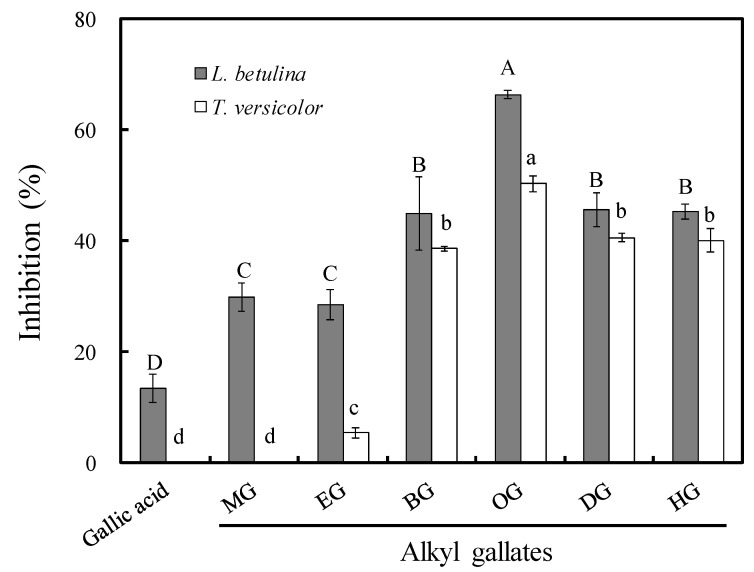
Effects of the alkyl chain length of gallates and gallic acid on antifungal activity against *L. betulina* and *T. versicolor* at the concentration of 100 μg/mL. MG: methyl gallate, EG: ethyl gallate, BG: butyl gallate, OG: octyl gallate, DG: decyl gallate, and HG: hexadecyl gallate. Values are expressed as the mean ± standard deviation (*n* = 3). Each bar with different letters is significantly different at *p* < 0.05.

An agar dilution test was performed to assess the antifungal activity of long-chain alkyl gallates against 100 μg/mL of *L. betulina* and *T. versicolor*. The antifungal indexes of gallic acid, MG, EG, BG, OG, DG, and HG were 13.4%, 29.9%, 28.6%, 44.9%, 66.4%, 45.6%, and 45.2%, respectively, against *L. betulina* and 0%, 0%, 5.4%, 38.6%, 50.3%, 40.6%, and 40.1%, respectively, against *T. versicolor (*Figure 1). Among them, OG had the best antifungal activity. Notably, the half maximal effective concentration (EC_50_) values of OG which were calculated by linear interpolation were 74.7 and 95.8 μg/mL against *L. betulina* and *T. versicolor*, respectively. This result indicated that the activity of gallates increased with the increase in alkyl chain length, reaching a maximum with OG, and then decreased as the chain length increased.

### 3.2. Antioxidant Activity and Ferrous Ion-Chelating Effect of Long-Chain Alkyl Gallates

**Figure 2:**
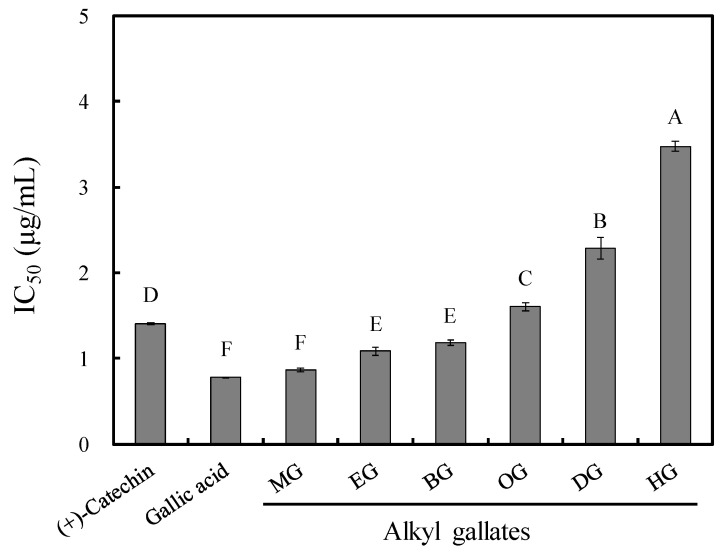
Effects of the alkyl chain length of gallates and gallic acid on DPPH radical-scavenging activity. MG: methyl gallate, EG: ethyl gallate, BG: butyl gallate, OG: octyl gallate, DG: decyl gallate, and HG: hexadecyl gallate. Values are expressed as the mean ± standard deviation (*n* = 3). Each bar with different letters is significantly different at *p* < 0.05.

The free radical-scavenging activity of the tested gallates was assessed using DPPH assay. As shown in Figure 2, all gallates showed strong DPPH radical-scavenging activity. The EC_50_ values of long-chain alkyl gallates slightly decreased with the increased chain length, possibly because gallates with more alkyl chains had a smaller proportion of pyrogallol moiety, leading to a reduction in antioxidant activity [7].

**Figure 3:**
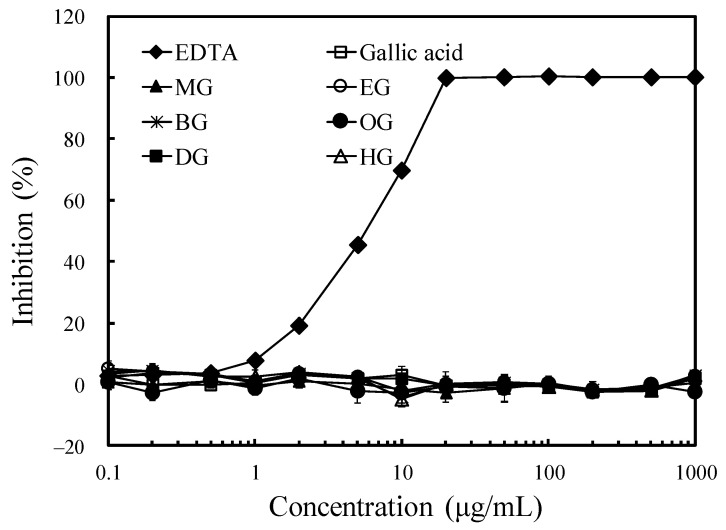
Effects of the alkyl chain length of gallates and gallic acid on ferrous ion-chelating ability. MG: methyl gallate, EG: ethyl gallate, BG: butyl gallate, OG: octyl gallate, DG: decyl gallate, and HG: hexadecyl gallate. Values are expressed as the mean ± standard deviation (*n* = 3).

Additionally, the ferrous ion-chelating activity of long-chain alkyl gallates is shown in Figure 3. Although the chelating ability of EDTA increased with the concentration, the long-chain alkyl gallates did not demonstrate any chelating ability. This result may be because the galloyl group was less capable of chelating iron and reduced iron binding [18]. However, when the free radical-scavenging and ferrous ion-chelating activities of long-chain alkyl gallates were compared with their antifungal activity, the results indicated that their action against white-rot fungi might not be completely related to their antioxidant activity or iron-binding capacity.

### 3.3. Effects of OG on the Laccase and Manganese Peroxidase Activities of L. betulina and T. versicolor

**Figure 4:**
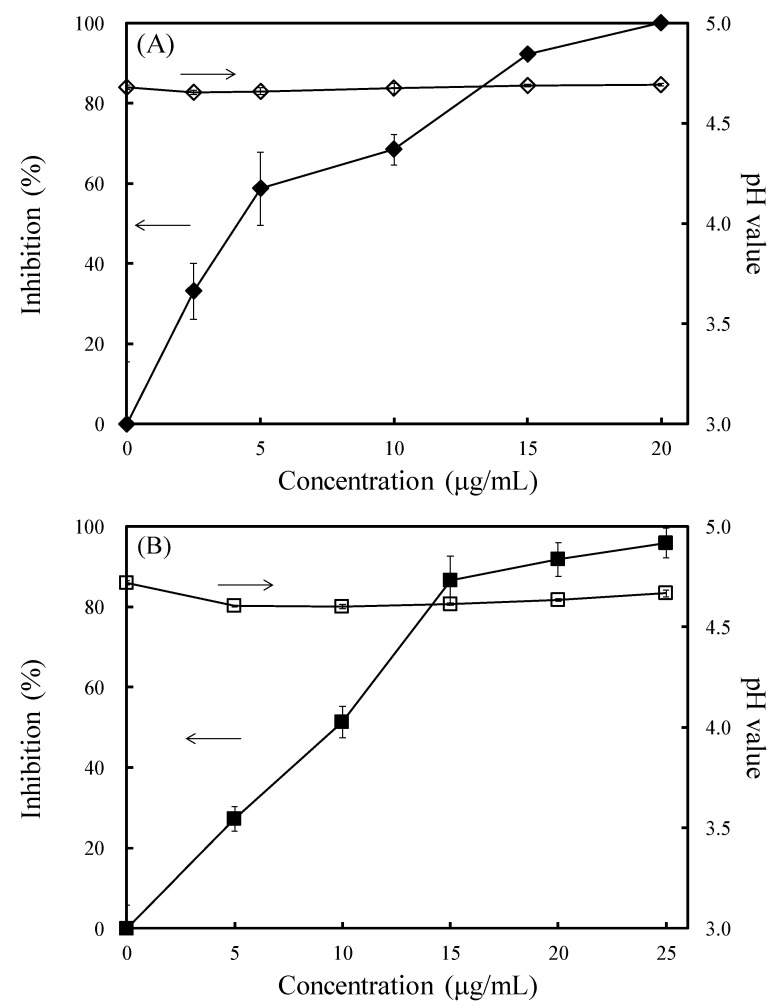
Effects of octyl gallate (OG) on the inhibition and medium acidification of *L. betulina* (**A**) and *T. versicolor* (**B**). Values are expressed as the mean ± standard deviation (*n* = 3). The arrows indicate the y-axis relative to the arrowed lines.

Among the gallate derivatives, OG had the best antifungal activity. Therefore, the further study of laccase and MnP were concentrated in OG. OG also inhibited fungal growth in the submerged cultivation test. As shown in Figure 4A,B, the antifungal indexes of OG against *L. betulina* at concentrations of 2.5, 5, 10, 15, and 20 μg/mL were 33.1%, 58.7%, 68.4%, 92.1%, and 99.9%, respectively, and against *T. versicolor* at concentrations of 5, 10, 15, 20, and 25 μg/mL were 27.3%, 51.3%, 86.6%, 91.8%, and 95.9%, respectively. However, the medium acidification exhibited no significant difference compared with the addition of OG (Figure 4A,B). To understand the effect of OG on laccase and MnP activities, the EC_50_ values (4.2 and 9.7 μg/mL against *L. betulina* and *T. versicolor*, respectively) of OG were selected for further study.

**Figure 5:**
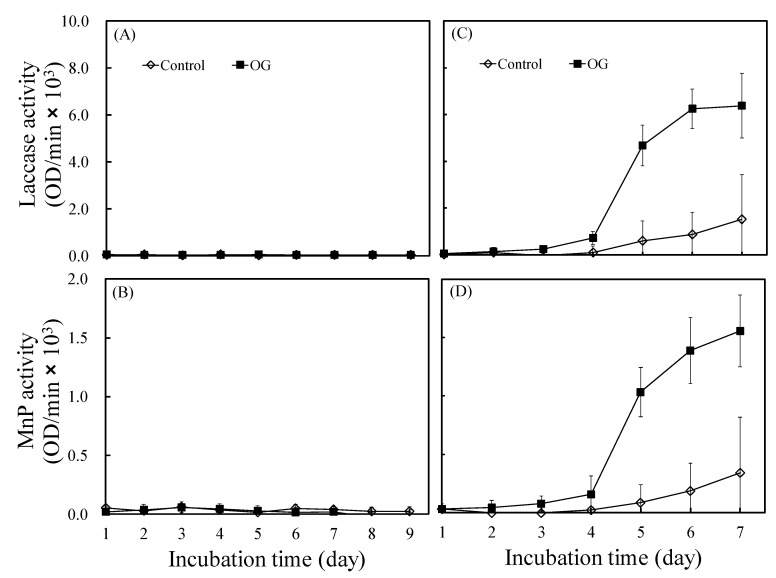
Effects of octyl gallate (OG) on the laccase and manganese peroxidase (MnP) activities of *L. betulina* at the concentration of 4 μg/mL (**A** and **B**, respectively) and *T. versicolor* at the concentration of 10 μg/mL (**C** and **D**, respectively). Values are expressed as the mean ± standard deviation (*n* = 3).

Figure 5 presents the laccase and MnP activities of *L. betulina* and *T. versicolor*. The rate and extent of laccase and MnP activities of *T. versicolor* were significantly higher than those of *L. betulina*, consistent with the reports of Sergentani et al. [19] and Alberts et al. [20]. Additionally, as shown in Figure 5A,B, neither enzyme of *L. betulina* was expressed when OG was added, consistent with the result reported by Tortella et al. [21]. By contrast, both enzymes of *T. versicolor* were significantly upregulated with the addition of OG, which may accelerate wood decay. This can be mainly attributed to laccase induction, which may play a defensive role through the catalyzation of polymerization or generation of oxidative stress by toxic aromatic compounds structurally related to lignin derivatives [22]. Additionally, the very close cooperation between superoxide dismutase and laccase may produce reactive oxygen species (ROS), which are fully involved in white-rot fungi-induced biodegradation [23]. Thus, OG may be different from typical antifungal agents, which exert their antifungal action through free radical scavenging, metal chelation, or enzyme inhibition. Its antifungal mechanisms may be worth studying. Proteomics is a powerful tool for studying changes in protein expression for pathogenic processes. Therefore, we performed proteomics analysis to identify the response of *L. betulina* and *T. versicolor* to the addition of OG.

### 3.4. Proteomics Analysis

**Figure 6:**
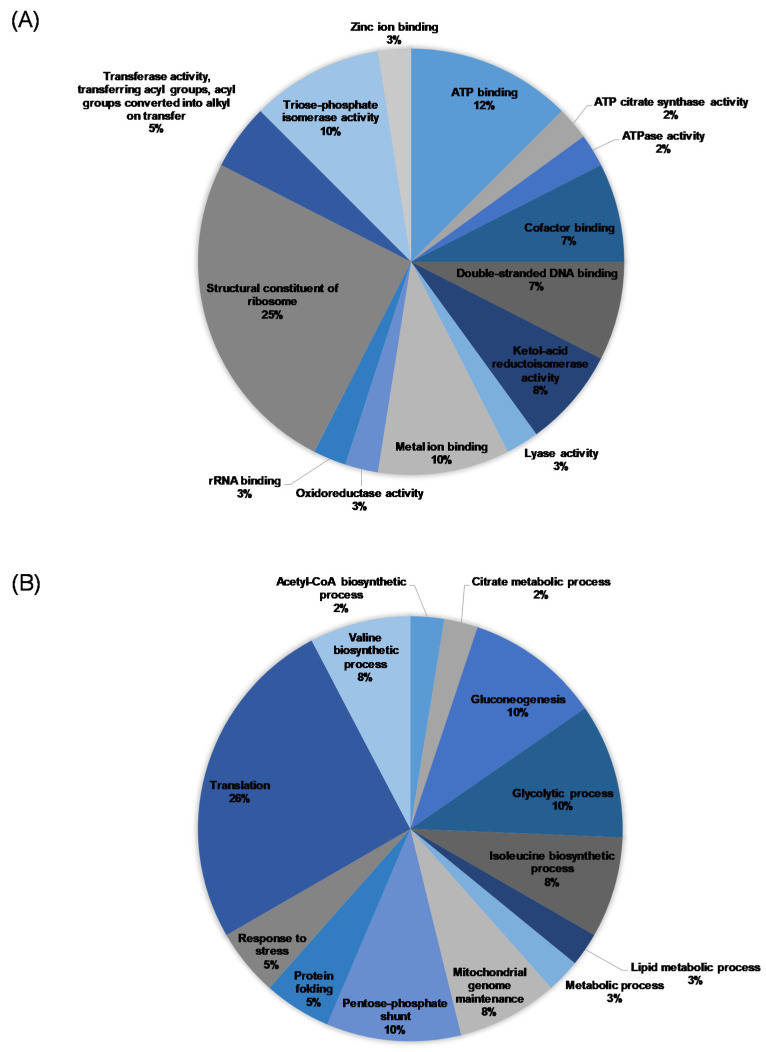
Classification of the differentially expressed proteins identified from *L. betulina*. Pie charts of the distribution of molecular function (**A**) and biological process (**B**).

The mycelial proteins were separated through electrophoresis and analyzed using MS/MS. The spectra generated were analyzed with Peaks 7.5 studio to identify the peptide sequences queried against the UniProt database. Next, a 2931 protein sequence was obtained, with 25 (Table 1 and Table 2) and 53 (Table 3 and Table 4) proteins that were considerably different before and after OG treatment in *L. betulina* and *T. versicolor*, respectively. As shown in Figure 6, OG treatment downregulated *L. betulina* proteins related to ATP binding, cofactor binding, the structural constituent of ribosome, and translation and upregulated those related to double-stranded DNA binding, ketol-acid reductoisomerase activity, triose-phosphate isomerase activity, gluconeogenesis, mitochondrial genome maintenance, pentose phosphate shunts, and the glycolytic, isoleucine biosynthetic, and valine biosynthetic processes.

ATPases are found in prokaryotes as well as in many eukaryotic plasma membranes and play a vital role in the transport of ions and phospholipids across the membrane [24]. Many studies have reported that gallate derivatives interact with microbial cell membranes, but how these compounds perturb cell membranes remains uncertain [25]. In the present study, the proteins related to ATP binding and ATPase activity were also downregulated, suggesting that the OG enters periplasm and inhibits transmembrane ATPase activity and further reduces the ATP level. While H^+^-ATPase is the most abundant plasma membrane protein, constituting over 20% of the total membrane protein in *Saccharomyces cerevisiae*, the conformation of the protein may be changed by OG, and the plasma membrane may lose its functioning conformations [11]. Moreover, mitochondrial oxidative phosphorylation was also required for ATP generation. Under normal conditions, protons return to the mitochondrial matrix through the F_0_ proton channel following the concentration gradient. However, adenosine diphosphate (ADP) was phosphorylated to generate ATP in the F_1_ domain of ATP synthase [26]. Therefore, OG not only caused mitochondrial dysfunction, which reduced the efficiency of energy-dependent processes, such as cell defenses against stress, but also prevented protons from returning to the mitochondrial matrix, leading to endogenous ROS generation.

Additionally, ATP citrate synthase and ATP citrate lyase isoform 2 related to ATP citrate synthase activity were downregulated with the increase in ketol-acid reductoisomerase and triosephosphate isomerase. In other words, OG may remodel the energy metabolism of *L. betulina* by lowering ATP levels and increasing glycolytic activity to compensate for the downregulation of aerobic pathways. Moreover, decreased efficiency of mitochondrial respiration in fluconazole-resistant strains reduces the ability of endogenous ROS to protect cells from being insulted by antifungal agents, ultimately reducing the resistance to fluconazole [26]. Thus, *L. betulina* may downregulate Krebs cycle (tricarboxylic acid cycle) activity to minimize the damage caused by endogenous ROS.

Ribosomal proteins play a vital role in monitoring and responding to cellular stress. After exposure to extracellular or intracellular stress, cells rapidly downregulate ribosomal RNA synthesis [27]. Additionally, the regulation of mRNA translation is an evolutionarily conserved mechanism for modulating longevity [28]. However, decreasing the abundance of 60S ribosomal subunits leads to increased replicative lifespan through the deletion of 60S-specific ribosomal processing factors or by the induction of endoplasmic reticulum stress [29]. In the present study, by regulating the cellular response to endoplasmic reticulum stress, *L. betulina* depleted 60S ribosomal subunits, thereby reducing the mycelial growth rate.

Heat shock proteins are highly conserved from bacteria to animals and function as molecular chaperones against stress by inhibiting denatured protein aggregation, helping damaged proteins to fold, or dissolving the denatured proteins [30]. Heat shock protein 90 is achieved by an inducible nitrogen-dependent promoter and causes decreased spore viability, decreased hyphal growth, and severe concomitant defects in germination and conidiation [31]. In this study, heat shock proteins 90 and 90-alpha were downregulated by OG, which may be a result of stress-responsive expression. Similar fungal responses have been reported with the addition of aromatic hydrocarbons [32].

**Figure 7:**
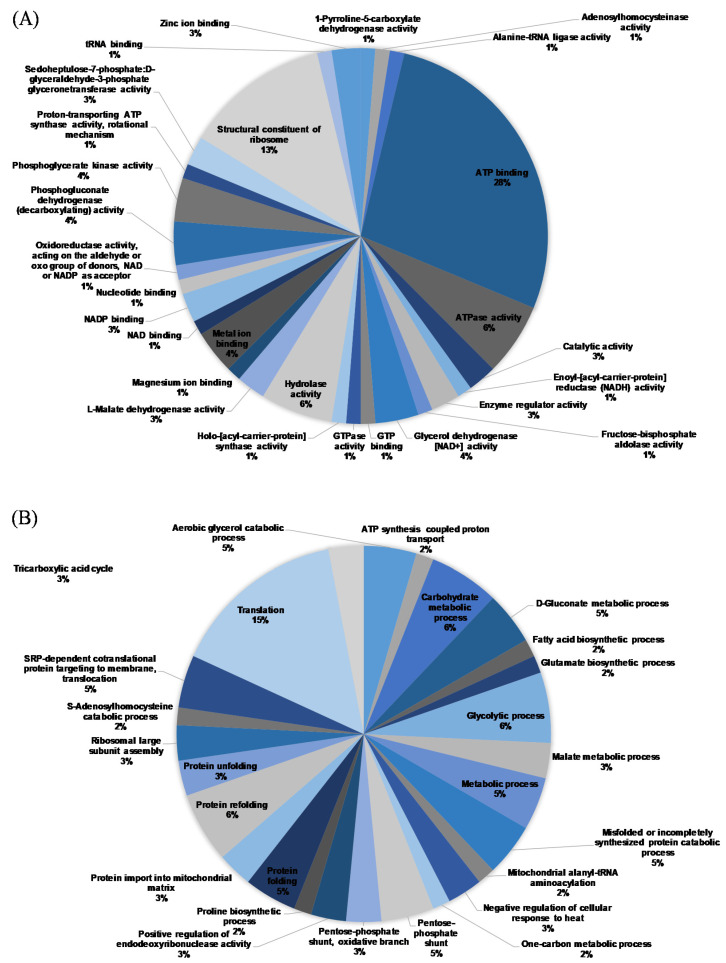
Classification of the differentially expressed proteins identified from *T. versicolor*. Pie charts of the distribution of molecular functions (**A**) and biological processes (**B**).

However, as shown in Figure 7, in *T. versicolor,* OG downregulated proteins related to protein folding and the aerobic glycerol catabolic, carbohydrate metabolic, glycolytic, metabolic, and misfolded or incompletely synthesized protein catabolic processes and upregulated those related to the _D_-gluconate metabolic process and translation. The trend of proteomics analysis in *T. versicolor* was similar to that in *L. betulina*, but the details are more complicated.

Similar to *L. betulina*, the proteins related to ATP binding and ATPase activity were also downregulated by OG, indicating that plasma membrane proteins may have been disrupted. However, glycerol dehydrogenase, malate dehydrogenase, phosphoglycerate kinase, and transaldolase were downregulated, whereas 6-phosphogluconate dehydrogenase was upregulated. This observation indicates the metabolic flux is redirected from glycolysis to the oxidative branch of the pentose phosphate pathway. The pentose phosphate pathway generates nicotinamide adenine dinucleotide phosphate (NADPH), a reducing agent [33]. NADPH serves as a cofactor for fatty acid synthesis and the recycling steps within the glutathione, thioredoxin, and peroxiredoxin systems, whose redox state control is crucial [34]. Overexpression of transaldolase reduces glucose 6-phosphate dehydrogenase and 6-phosphogluconate dehydrogenase activities, thus reducing NADPH and glutathione production and increasing oxidative stress. Suppression of transaldolase results in the opposite effects [35].

However, 60S ribosomal proteins of *T. versicolor* were also downregulated by OG, and 40S ribosomal proteins were upregulated. The results revealed that the 40S preinitiation complexes accumulated in half-mer polyribosomes in the absence of sufficient 60S subunits [36]. Moreover, similar to *L. betulina*, heat shock protein 70 and proteins related to actin were also downregulated in *T. versicolor* by OG. Unlike heat shock protein 90, heat shock protein 70 refolds and maintains denatured proteins in vitro, but it also has antiapoptotic activity [37].

Thus, proteomic analysis revealed that OG may act similarly to surfactants by in-hibiting ATPase activity, leading to mitochondrial dysfunction and endogenous ROS production. However, after OG treatment, there was no significant difference in the proteins related to laccase and MnP. After exposure to endogenous ROS, cells rapidly downregulated the synthesis of 60S ribosomal subunits, leading to an increased replicative lifespan, thereby reducing the mycelial growth rate. Additionally, *L. betulina* and *T. versicolor* also remodeled their energy metabolism in response to low ATP levels and endogenous ROS. In *L. betulina*, the ATP citrate synthase activity was downregulated and glycolytic activity was upregulated in response to low ATP levels. However, *T. versicolor* not only reduced its aerobic pathway activity but also upregulated 6-phosphogluconate dehydrogenase and transaldolase, resulting in NADPH generation to minimize the damage from endogenous ROS. 

## 4. Conclusions

To develop effective and environmentally friendly wood preservatives, this study investigated the antifungal activity and mechanism of a series of long-chain alkyl gallates (3,4,5-trihydroxybenzoates) against the white-rot fungi *L. betulina* and *T. versicolor*. The results revealed that octyl gallate (OG) had the best antifungal activity, with EC_50_ values of 74.65 μg/mL (*L. betulina*) and 95.80 μg/mL (*T. versicolor*). Unexpectedly, however, the activity of laccase and MnP in *T. versicolor* was upregulated with the addition of OG. To understand how white-rot fungi respond to OG, we conducted proteomics analysis to identify the changes in proteins. We found that OG impeded the metabolic flexibility of *L. betulina* and *T. versicolor*. After OG treatment, ATP citrate synthase activity was downregulated and glycolytic activity was upregulated in *L. betulina*, while *T. versicolor* not only reduced aerobic pathway activity but also redirected the pathway to the oxidative branch of the pentose phosphate pathway. Furthermore, OG may inhibit ATPase activity, causing mitochondrial dysfunction and endogenous ROS production. Endogenous ROS also caused ATP-dependent protein (heat shock proteins and 60S ribosomal protein) folding to function suboptimally, leading to membrane damage. Therefore, OG can be a potent inhibitor of white-rot fungi, although further structural optimization and pharmacological investigations are warranted.

## Figures and Tables

**Table 1:** Proteins of *Lenzites betulina* that were upregulated due to the addition of octyl gallate.

Protein Name	Accession No.	pI	MW	GO	Expression Ratio
60S ribosomal protein L9-B (Schizosaccharomyces pombe 972h-)	A0A0K6FS64_9HOMO	9.38	21,383.81	-	3.92
Ketol-acid reductoisomerase, mitochondrial	W4KC19_9HOMO	8.85	44,553.29	-	2.17
Ketol-acid reductoisomerase, mitochondrial	A0A0C3DUH5_9HOMO	8.91	44,841.78	-	2.04
Ketol-acid reductoisomerase, mitochondrial	A0A060SWV9_PYCCI	8.98	44,706.68	-	1.98
Triosephosphate isomerase	R4I570_9AGAR	5.70	26,921.71	-	3.89
Triosephosphate isomerase	A0A0C3PQJ3_PHLGI	5.50	26,627.37	-	3.46
Triosephosphate isomerase	A0A151V9C9_HYPMA	5.97	26,765.62	-	3.46
Triosephosphate isomerase	S8FUL8_FOMPI	6.00	26,850.73	-	3.46
Uncharacterized protein	A0A0C3S325_PHLGI	11.17	20,963.75	0006412	3.84

pI: theoretical isoelectric point, MW: molecular weight, and GO: gene ontology.

**Table 2:** Proteins of *Lenzites betulina* that were downregulated due to the addition of octyl gallate.

Protein Name	Accession No.	pI	MW	GO	Expression Ratio
60S ribosomal protein L12	A0A165F4X5_9APHY	9.19	17,522.26	0006412	0.09
60S ribosomal protein L12	A0A165RLF8_9APHY	9.06	17,510.30	0006412	0.09
60S ribosomal protein L12	A0A1C7LQZ3_GRIFR	9.35	17,364.10	-	0.09
60S ribosomal protein L12	B8P7S1_POSPM	9.19	17,550.31	0006412	0.09
ATP citrate lyase isoform 2	A0A0D7B0G9_9HOMO	6.55	125,756.11	-	0.11
ATP citrate synthase	A0A165GIY9_9APHY	8.33	125,805.87	-	Control only
GroES-like protein	A0A166C2Q9_9HOMO	6.37	37,305.72	-	0.25
Heat shock protein 90	R7S7P6_TRAVS	4.95	79,624.25	0006457 0006950	0.39
Heat shock protein HSP 90-alpha	A0A0B7FTU3_THACB	4.94	79,834.83	-	0.36
Mitochondrial glycoprotein	R7SPA0_DICSQ	4.67	29,577.92	-	0.25
Uncharacterized protein	J4HUC5_9APHY	9.59	37,362.90	0006412	0.09
Uncharacterized protein	M2R3S2_CERS8	9.06	17,655.46	0006412	0.09
Uncharacterized protein	A0A060S428_PYCCI	7.63	107,357.17	-	0.21
Uncharacterized protein	A0A0C3P9N6_PHLGI	9.32	17,499.22	0006412	0.09
Uncharacterized protein	K5UVC6_PHACS	9.19	17,396.05	0006412	0.09
Uncharacterized protein	S8FGW3_FOMP	9.06	17,496.28	0006412	0.09

pI: theoretical isoelectric point, MW: molecular weight, and GO: gene ontology.

**Table 3:** Proteins of *Trametes versicolor* that were upregulated due to the addition of octyl gallate.

Protein Name	Accession No.	pI	MW	GO	Expression Ratio
40S ribosomal protein	A0A146HFY9_9AGAR	10.20	17,473.48	0006412	4.99
40S ribosomal protein S15	A0A0B7FZY5_THACB	10.24	17,610.61	0006412	3.78
40S ribosomal protein S15	A0A151VTC7_HYPMA	10.31	17,514.54	0006412	3.78
40S ribosomal protein S15	L8X3B3_THACA	9.90	16,454.27	0006412	3.78
6-phosphogluconate dehydrogenase, decarboxylating	A0A0C2XM62_AMAMU	6.35	53,162.64	-	2.43
6-phosphogluconate dehydrogenase, decarboxylating	A0A0D7B7I4_9HOMO	5.79	53,373.75	0019521 0009051	3.72
6-phosphogluconate dehydrogenase, decarboxylating	A0A146HJH8_9AGAR	7.09	53,411.28	-	2.37
ATP synthase subunit alpha	K5VTN2_PHACS	9.15	58,843.66	0015986	1.99
Chaperonin GroL	A0A165LVH4_9APHY	5.79	62,855.91	-	2.14
Heat shock protein	A8PB53_COPC7	5.62	63,344.06	-	2.20
Uncharacterized protein	A0A060S4R5_PYCCI	9.04	19,588.59	0006412	2.84
Uncharacterized protein	A0A060SGS2_PYCCI	9.74	13,299.48	0006412	7.99
Uncharacterized protein	R7SX26_DICSQ	10.51	17,474.38	-	4.66

pI: theoretical isoelectric point, MW: molecular weight, and GO: gene ontology.

**Table 4:** Proteins of *Trametes versicolor* that were downregulated due to the addition of octyl gallate.

Protein Name	Accession No.	pI	MW	GO	Expression Ratio
60S ribosomal protein L3	B8PIG2_POSPM	10.26	4,3761.83	-	0.45
60S ribosomal protein L4/L1/L2	A0A165ZAV7_9HOMO	11.12	40,412.82	0006412	0.23
AAA ATPase	A0A165B9Y7_9APHY	4.99	89,912.01	-	0.46
Actin 1	R7SI12_DICSQ	5.44	41,692.58	-	0.37
Actin-1	A0A1C7LPB9_GRIFR	5.35	42,816.81	-	0.37
Adenosylhomocysteinase	M2QCQ6_CERS8	5.74	47,267.26	-	0.42
Alanine--tRNA ligase	A0A060SL88_PYCCI	5.75	106,172.69	-	0.46
Beta-actin	V5W5W4_GANLU	5.44	41,520.41	-	0.37
Beta-actin-like protein	M2QI81_CERS8	5.44	41,692.58	-	0.37
Delta-1-pyrroline-5-carboxylate dehydrogenase 1	A0A165ZHP2_9HOMO	6.48	59,553.60	-	0.32
Fructose 1,6-bisphosphate aldolase	A0A1B7N8B6_9HOMO	6.02	38,850.08	-	Control only
Glycerol dehydrogenase	R7S6H2_TRAVS	5.30	42,121.11	-	0.46
Glycerol dehydrogenase	R7SKI4_DICSQ	5.58	40,618.43	-	0.52
Heat shock cognate 70	A0A0H2RYR8_9HOMO	5.13	70,997.65	1900035	0.45
Heat shock protein 70	R7SN52_DICSQ	5.64	71,837.59	-	0.48
Malate dehydrogenase	A0A165P5J0_9HOMO	6.45	33,510.44	0005975 0006108 0006099	0.31
Malate dehydrogenase	S7RXX1_GLOTA	9.05	35,546.85	0005975 0006108 0006099	0.31
Phosphoglycerate kinase	A0A0C2XI58_AMAMU	5.97	44,767.53	0006096	0.16
Phosphoglycerate kinase	A0A0C3S5Q2_PHLGI	5.99	44,470.21	0006096	0.12
Phosphoglycerate kinase	A0A146IDB4_9AGAR	6.15	44,134.66	-	Control only
Putative carboxymethylenebutenolidase	A0A137QS07_9AGAR	6.75	28,102.12	-	0.33
Related to HSP70 heat shock protein 70 (Hsp70)	G4T8Z4_SERID	5.14	70,572.25	0006515 0006457 0006616	0.42
Transaldolase	A0A060SMY4_PYCCI	6.19	36,149.43	0005975 0006098	0.35
Transaldolase	R7S6N3_TRAVS	7.64	28,010.29	0005975 0006098	0.42
Transketolase-like protein 2	A0A1C7M6L6_GRIFR	6.33	64,753.10	-	0.15
Uncharacterized protein	A0A060S8Y5_PYCCI	5.03	89,836.05	-	0.52
Uncharacterized protein	J4G7W8_9APHY	4.97	89,888.93	-	0.48
Uncharacterized protein	J4GJQ5_9APHY	5.56	72,198.74	-	0.51
Uncharacterized protein	A0A060SCB9_PYCCI	5.92	433,211.19	-	0.35
Uncharacterized protein	A0A060SEM9_PYCC	5.21	40,163.80	-	0.45
Uncharacterized protein	A0A060SFD3_PYCCI	5.13	70,262.90	0006515 0006457 0006616	0.44
Uncharacterized protein	A0A060SNB2_PYCCI	6.51	92,680.46	-	0.34
Uncharacterized protein	A0A060SR94_PYCCI	5.97	69,483.20	0008152	0.27
Uncharacterized protein	A0A0C3NYK6_PHLGI	5.37	41,591.47	-	0.17
Uncharacterized protein	A0A0D2PLJ0_9AGAR	6.31	30,884.26	-	0.34
Uncharacterized protein	J4HZZ4_9APHY	9.79	106,199.49	-	0.46
Uncharacterized protein	S8ET35_FOMPI	5.10	71,000.55	1900035	0.46
Unplaced genomic scaffold CY34scaffold_2, whole genome shotgun sequence	A0A0D0AGV4_9HOMO	5.12	70,384.87	0006515 0006457 0006616	0.37

pI: theoretical isoelectric point, MW: molecular weight, and GO: gene ontology.

## Data Availability

The data that support this study are available from the corresponding author upon reasonable request.
